# A Comprehensive Larval microRNA Atlas of *Hyphantria cunea* Identifies Candidate miRNAs and Potential Molecular Targets for Green Pest Management

**DOI:** 10.3390/ijms27093884

**Published:** 2026-04-27

**Authors:** Yanqin Zhu, Kai Tang, Mao Lin, Shuaishuai Fanji, Shouke Zhang

**Affiliations:** State Key Laboratory for Development and Utilization of Forest Food Resources, Zhejiang A&F University, Hangzhou 311300, China

**Keywords:** *Hyphantria cunea*, larvae, microRNA, target genes, RNAi

## Abstract

*Hyphantria cunea* (Drury) causes extensive ecological damage primarily during its larval stages, characterized by voracious feeding and rapid dispersal. Given that conventional dsRNA-mediated RNA interference (RNAi) is generally recalcitrant in Lepidoptera, endogenous microRNAs (miRNAs) may represent an additional class of regulatory molecules worthy of systematic investigation. In this study, we utilized high-throughput sequencing to construct nine comprehensive miRNA libraries across three critical developmental milestones (three biological replicates per instar): the 1st, 4th, and 7th instars (L1, L4, and L7). A total of 1667 miRNA entries were catalogued, including 1080 known and 587 bioinformatically predicted, as yet unvalidated novel miRNA candidates. Comparative transcriptomic analysis revealed 52 differentially expressed miRNAs with significant stage-dependent profiles, with the most pronounced divergence observed between the L1 and L7 groups. Bioinformatic prediction identified 16,784 non-redundant target genes. GO and KEGG enrichment analyses indicated that the predicted target genes of these differentially expressed miRNAs were enriched in developmental and metabolic categories, including cellular development, protein digestion, and nutrient absorption, suggesting that these miRNAs may be associated with tissue remodeling and larval developmental transitions. Collectively, our findings expand the currently available miRNA resource for *H. cunea* and define stage-associated miRNA expression patterns during larval development. Rather than establishing direct functional roles, this work provides a framework and candidate molecules for future design of RNAi-based biopesticides.

## 1. Introduction

*Hyphantria cunea*, a globally invasive quarantine pest, is characterized by its extraordinary ecological plasticity and profound invasion potential [1]. Since its introduction into China, this polyphagous species has successfully colonized diverse ecological niches; its broad host spectrum, voracious feeding habits, and rapid population expansion have resulted in staggering economic and ecological consequences [2]. The larval stage represents the pivotal phase for intensive defoliation and nutrient acquisition, serving as a determinant of population fitness and a strategic window for pest intervention [3]. Notably, early larvae (1st-3rd instars) typically remain aggregated, whereas dispersal begins from the 4th instar onward [4]. While chemical insecticides have historically been the mainstay for suppressing *H. cunea* populations, their prolonged and intensive application has inevitably led to environmental degradation, biodiversity erosion, and the escalation of insecticide resistance [5,6]. Consequently, there is an imperative need to develop highly efficacious, precise, and eco-compatible management technologies, which remains a top priority in current integrated pest management (IPM) research for *H. cunea* [7].

Recently, RNA-based biopesticides leveraging RNA interference (RNAi) technology have emerged as a pivotal frontier for the sustainable management of agricultural and forestry pests [8]. RNAi is a conserved biological mechanism through which double-stranded RNA (dsRNA) or small interfering RNA (siRNA) mediates the sequence-specific degradation or translational repression of target mRNAs [9]. This mechanism has evolved into a robust tool for functional genomics and holds immense potential for pest and disease management in agriculture and forestry [10]. However, the practical deployment of RNA-based pest control still faces substantial challenges, including variable RNAi efficiency among taxa, delivery constraints, molecular stability, and biosafety assessment [11,12,13]. In insects, three major small RNA pathways are typically categorized: siRNAs, piRNAs, and miRNAs [9]. Among these, dsRNA-induced gene silencing predominantly relies on the siRNA pathway, constituting the principal route in current RNAi research and applications [14]. However, RNAi efficiency varies markedly among insect taxa [15,16,17]. In contrast to the relatively efficient and stable dsRNA responses reported in Coleoptera, lepidopteran insects generally exhibit weak and inconsistent responses to exogenous dsRNA [15,18], likely owing to multiple physiological and cellular barriers that constrain RNAi efficiency [19,20]. Previous studies have demonstrated that exogenous dsRNA is readily degraded within the alkaline gut environment and associated nucleases of lepidopteran insects, which limits the application of conventional dsRNA-based approaches in lepidopteran pest control [21,22]. Consequently, identifying novel RNA-interference molecules and potential targets in Lepidoptera represents a vital direction for expanding RNAi applications [16]. Recent miRNA-based studies in lepidopteran pests, including *Plutella xylostella* [23], *Helicoverpa armigera* [24], *Chilo suppressalis* [25], and *Tuta absoluta* [26], have further validated the application potential of miRNAs, highlighting the potential relevance of miRNAs as candidate molecules for future RNA-based control research [27].

MicroRNAs (miRNAs) are endogenous, non-coding small RNAs, typically averaging 22 nucleotides (nt) in length [28,29]. In animals, miRNAs typically exhibit imperfect complementarity with the 3′ untranslated regions (3′UTRs) of target mRNAs, thereby modulating gene expression at the post-transcriptional level [30]. Advances in small RNA sequencing have facilitated the construction of small RNA (sRNA) libraries, which has become a standardized approach for miRNA identification, expression profiling, and novel miRNA prediction, thereby enabling the systematic screening of candidate molecules. Since the pioneering discovery of the developmental regulator lin-4 in nematodes [31], miRNA research has expanded across diverse insect taxa; miRNA-mediated regulation is now known to extensively govern fundamental biological processes, including development, metamorphosis, reproduction, diapause, and stress resistance [32,33,34]. To date, research on *H. cunea* management has predominantly focused on chemical control [35] and the application of sex pheromones [36], while mechanistic studies on miRNAs in this species remain comparatively sparse. Given that the larval stage is the critical period for nutritional acquisition and damage manifestation in *H. cunea*, the systematic identification and profiling of larval miRNAs will not only broaden the miRNA repertoire of this species but also provide a fundamental framework for identifying instar-associated candidate miRNAs and prioritizing putative targets for future functional evaluation in RNAi-based pest management research.

Based on these considerations, the present study focused on three representative larval instars of *H. cunea* (L1, L4, and L7). By employing high-throughput small RNA sequencing, we generated comprehensive miRNA datasets, characterized known miRNAs, and identified novel miRNA candidates. Through integrated bioinformatic pipelines, we identified miRNAs exhibiting significant differential expression profiles across instars. Furthermore, we predicted their target genes and performed GO and KEGG enrichment analyses to elucidate the putative biological functions and regulatory pathways associated with these targets. This study aimed to systematically dissect the temporal miRNA expression patterns during *H. cunea* larval development, thereby substantially expanding the miRNA repertoire of this species.

## 2. Results

### 2.1. High Sequencing Quality and Distinct Small RNA Length Distribution Patterns Among Larval Instars

Total RNA was extracted from 1st-, 4th-, and 7th-instar larvae of *H. cunea* to construct three miRNA libraries, each with three biological replicates (L1_1, L1_2, L1_3, L4_1, L4_2, L4_3, L7_1, L7_2, and L7_3). Illumina high-throughput sequencing generated 14,927,824, 30,978,208, and 15,973,602 raw reads from the three 1st-instar libraries; 15,454,540, 15,475,532, and 16,405,506 raw reads from the three 4th-instar libraries; and 16,284,405, 16,339,754, and 18,623,270 raw reads from the three 7th-instar libraries (Table 1). All raw reads have been deposited in the Genome Sequence Archive (GSA) of the National Genomics Data Center (NGDC). After quality control, the three 1st-instar libraries yielded 7,233,279, 18,113,019, and 9,341,463 clean reads; the three 4th-instar libraries yielded 8,097,775, 7,341,611, and 6,689,861 clean reads; and the three 7th-instar libraries yielded 9,106,967, 8,783,918, and 8,518,726 clean reads (Table 1 and Table S2).

**Figure 1:**
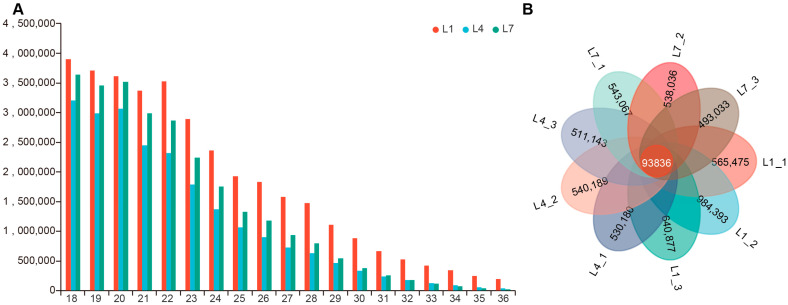
Length distribution of small RNAs and overlap of clean sequences among miRNA libraries from different larval instars of *Hyphantria cunea*. (**A**) Length distribution of total clean reads (18–36 nt) in the three larval libraries (L1, L4, and L7). Different colors indicate the three libraries. The x-axis indicates sequence length, and the y-axis indicates sequence abundance. Each bar represents the number of reads within the corresponding length class. This panel reflects the overall size distribution of retained small-RNA reads rather than annotated mature miRNAs only. (**B**) Petal diagram showing the overlap of deduplicated sequences among the nine samples (L1_1-L1_3, L4_1-L4_3, and L7_1-L7_3). Each petal represents one sample, and the number within each petal indicates the number of deduplicated sequences unique to that sample. The number in the central overlapping region indicates the number of deduplicated sequences shared by all samples. Note: L1, L4, and L7 represent samples collected from the first-, fourth-, and seventh-instar larvae, respectively.

Length-distribution analysis of retained clean reads ranging from 18 to 36 nt after adapter trimming and length filtering showed that the overall small RNA profiles of the 4th- and 7th-instar libraries were similar and displayed two major peaks, with the dominant peak at 18 nt and a second peak at 20 nt. By contrast, the 1st-instar libraries showed four peaks, with the major peak at 18 nt and additional peaks at 19, 20, and 22 nt (Figure 1A). After deduplication of clean reads, 659,311, 1,078,229, and 734,713 unique reads were obtained from the three 1st-instar libraries; 624,016, 634,025, and 604,979 unique reads were obtained from the three 4th-instar libraries; and 636,903, 631,872, and 586,869 unique reads were obtained from the three 7th-instar libraries (Figure 1B).

To further characterize the genomic distribution of small RNAs, we aligned the clean sequences from the three libraries to the *H. cunea* genome using miRDeep2. The proportions of reads mapped to the genome were 59.10%, 55.33%, and 48.79% for the three 1st-instar replicates; 84.55%, 54.93%, and 55.32% for the three 4th-instar replicates; and 65.13%, 72.20%, and 72.90% for the three 7th-instar replicates (Table 1).

### 2.2. miRNA Identification Across Larval Instars Revealed Both Shared and Highly Abundant Known and Novel miRNAs

**Figure 2:**
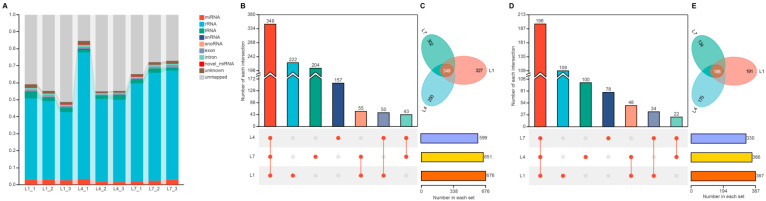
Overview of small RNA-sequencing data and miRNA identification in *Hyphantria cunea* larvae. (**A**) Stacked bar chart showing the composition and relative abundance of small RNA categories in first-instar (L1), fourth-instar (L4), and seventh-instar (L7) larvae of *H. cunea*, based on the annotated clean reads from each library. Different colors indicate different classes of small RNAs. Per-library category distributions are provided in Table S3. (**B**) UpSet plot showing the intersection of known miRNAs among the three larval instars. The bar plot at the lower right indicates the total number of miRNAs detected in each instar group. The matrix at the lower left shows the intersections among groups; colored dots indicate the corresponding intersections, whereas gray dots indicate the absence of intersection. The upper bar plot shows the number of miRNAs in each intersection set. (**C**) Petal diagram showing the shared and unique known miRNAs among the three larval instars. (**D**) UpSet plot showing the intersection of bioinformatically predicted novel miRNAs among the three larval instars. The bar plot at the lower right indicates the total number of miRNAs detected in each instar group. The matrix at the lower left shows the intersections among groups; colored dots indicate the corresponding intersections, whereas gray dots indicate the absence of intersection. The upper bar plot shows the number of miRNAs in each intersection set. (**E**) Petal diagram showing the shared and unique bioinformatically predicted novel miRNAs among the three larval instars.

Using the miRBase database, we annotated the clean reads obtained from the three libraries. These small RNA sequences were classified as miRNA, rRNA, tRNA, snRNA, snoRNA, exon, intron, novel_miRNA, unknown, or unmapped (Figure 2A and Table S3). Small-RNA sequences from the libraries were aligned against miRBase by BLAST (v2.16.0) to identify known miRNAs, and novel miRNAs were predicted using mireap. In total, 1080 known miRNAs and 587 bioinformatically predicted novel miRNAs (Tables S4 and S5) were identified from the three miRNA libraries (1st-, 4th-, and 7th-instar larvae). Among the known miRNAs, 676, 599, and 651 were detected in the 1st-, 4th-, and 7th-instar libraries, respectively (Figure 2B). Among the bioinformatically predicted novel miRNAs, 387, 366, and 330 were detected in the corresponding libraries (Figure 2D). Notably, 349 known miRNAs and 196 bioinformatically predicted novel miRNAs were expressed across all three larval instars (Figure 2C,E).

Across the three instar-specific miRNA libraries of *H. cunea* larvae, the most highly expressed known miRNA was animal-miR-2766, followed by animal-miR-276-3p_2, animal-miR-1, animal-miR-278-3p_2, and animal-miR-750_5 (Table 2). Among the bioinformatically predicted novel miRNAs, the most highly expressed was novel-m0029-3p, followed by novel-m0026-3p, novel-m0034-3p, novel-m0021-3p, and novel-m0025-3p (Table 3).

### 2.3. Differentially Expressed miRNAs Varied Markedly Among Instars and Exhibited Stage-Dependent Expression Patterns

To identify differentially expressed miRNAs among instars and reveal their potential regulatory patterns, we compared miRNA expression profiles among the three larval instars (1st, 4th, and 7th) using DESeq2, with thresholds of |log_2_(fold change)| > 1 and *p*-value < 0.05. Three pairwise comparison groups were defined: L1_vs_L4, L1_vs_L7, and L4_vs_L7. The numbers of differentially expressed miRNAs in these three comparisons were 16, 43, and 18, respectively, and no differentially expressed miRNA was shared among all three groups. Among these comparisons, L1_vs_L7 contained the largest number of differentially expressed miRNAs.

**Figure 3:**
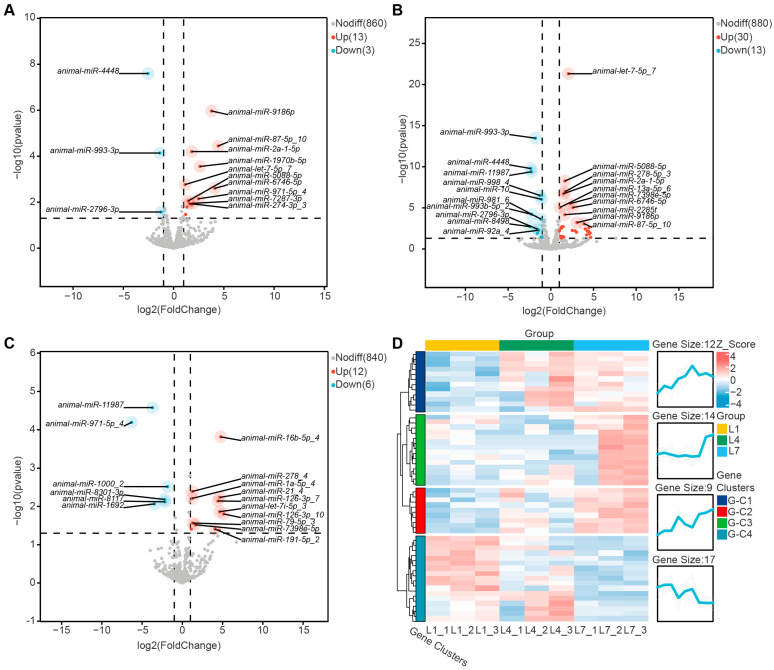
Identification and clustering of differentially expressed miRNAs among larval instars of *Hyphantria cunea*. (**A**–**C**) Volcano plots showing differentially expressed miRNAs in the pairwise comparisons between the first and fourth instars (**A**), the first and seventh instars (**B**), and the fourth and seventh instars (**C**). The x-axis represents log_2_(fold change), and the y-axis represents −log_10_(*p*-value). The vertical dashed lines denote the fold-change threshold, and the horizontal dashed line denotes the significance threshold. Colors indicate upregulated, downregulated, and non-significant miRNAs. Representative miRNAs with the highest statistical significance among the upregulated and downregulated sets are labeled according to *p*-value ranking, with the top 10 miRNAs from each set shown in each comparison. (**D**) Hierarchical clustering heatmap and expression trend plot of differentially expressed miRNAs across samples. The plots were generated using TPM values after Z-score normalization. In the heatmap, each column represents one sample, and colors indicate relative expression levels, with red representing relatively high expression and blue representing relatively low expression. The dendrogram on the left groups miRNAs with similar expression patterns. The trend plot on the right shows the expression trajectory of each cluster across samples, and the red line indicates the mean expression value of all miRNAs within that cluster.

In all pairwise comparisons, the number of upregulated miRNAs exceeded that of downregulated miRNAs. Specifically, 13 miRNAs were significantly upregulated and 3 were significantly downregulated in the comparison between 1st- and 4th-instar larvae (L1_vs_L4; Figure 3A). In the comparison between 1st- and 7th-instar larvae (L1_vs_L7), 30 miRNAs were significantly upregulated and 13 were significantly downregulated (Figure 3B). In the comparison between 4th- and 7th-instar larvae (L4_vs_L7), 12 miRNAs were significantly upregulated and 6 were significantly downregulated (Figure 3C). Full differential expression statistics for all miRNAs, including fold change, *p*-value, and adjusted *p*-value, are provided in Tables S6–S8.

Hierarchical clustering based on miRNA expression levels grouped these miRNAs into four clusters according to similarity in expression pattern. In G-C1 (*n* = 12), expression was low in the 1st instar, increased markedly in the 4th instar, and remained high in the 7th instar. G-C2 (*n* = 9) showed a progressive increase across development, with a 1st- to 4th- to 7th-instar upward trajectory. G-C3 (*n* = 14) showed low expression in the 1st and 4th instars but was specifically upregulated in the 7th instar. By contrast, G-C4 (*n* = 17) exhibited a continuous downward trend opposite to that of G-C2. These clusters describe shared expression trajectories among differentially expressed miRNAs across instars. Overall, biological replicates were highly consistent within each group, indicating good reproducibility of the observed instar-dependent dynamics (Figure 3D).

### 2.4. Target Genes of Differentially Expressed miRNAs Were Enriched in Development-And Metabolism-Related Pathways and Showed Functional Divergence Among Comparisons

Target genes of the differentially expressed miRNAs from *H. cunea* larvae at the three instars (1st, 4th, and 7th) were predicted using MiRanda, RNAhybrid, and TargetScan. Only targets predicted by at least two of the three programs were retained for downstream analysis. In total, 16,784 non-redundant target genes were predicted for the 52 differentially expressed miRNAs. The numbers of predicted target genes varied across individual miRNAs (Table S9).

**Figure 4:**
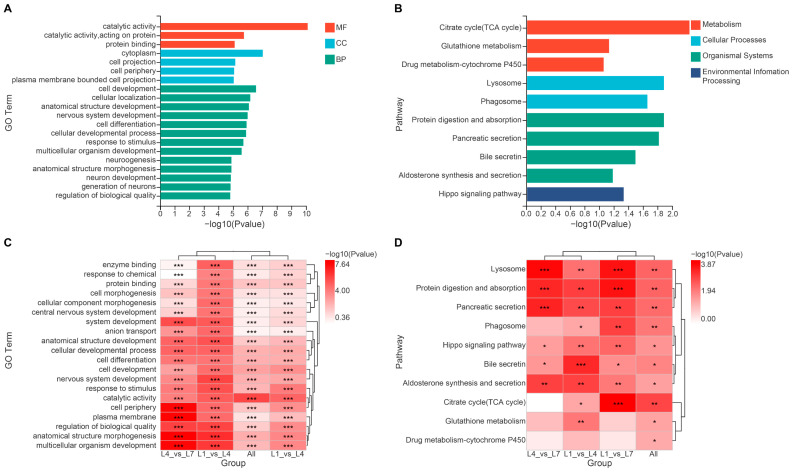
GO and KEGG enrichment analyses of candidate target genes of differentially expressed miRNAs. (**A**) Bar plot showing enriched GO terms for the candidate target genes of differentially expressed miRNAs. The x-axis represents -log_10_(*p*-value), and the y-axis represents enriched GO terms. Different colors indicate the three major GO categories: biological process (BP), cellular component (CC), and molecular function (MF). (**B**) Bar plot showing enriched KEGG pathways for the candidate target genes of differentially expressed miRNAs. The x-axis represents –log_10_(*p*-value), and the y-axis represents enriched KEGG pathways. Different colors indicate pathway categories, including Metabolism, Cellular Processes, Organismal Systems, and Environmental Information Processing. (**C**) Heatmap of GO enrichment results. The pairwise comparison groups (L1_vs_L4, L1_vs_L7, and L4_vs_L7) and the combined set (All) are compared for enrichment significance across representative GO terms. The x-axis represents comparison groups, and the y-axis represents GO terms. Colors indicate the significance level. *, **, and *** indicate *p* < 0.05, *p* < 0.01, and *p* < 0.001, respectively. Blank cells indicate that the corresponding term was not enriched or did not pass the filtering threshold in that comparison group. (**D**) Heatmap of KEGG enrichment results. The pairwise comparison groups (L1_vs_L4, L1_vs_L7, and L4_vs_L7) and the combined set (All) are compared for enrichment significance across representative KEGG pathways. Colors and asterisks are interpreted as described in (**C**).

**Figure 5:**
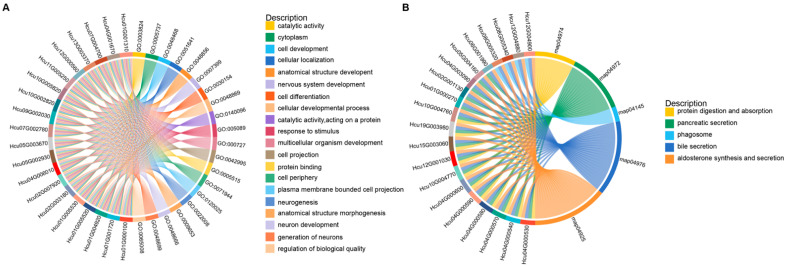
Chord diagrams showing the relationships between enriched functional categories and candidate target genes. (**A**) GO chord diagram showing the relationships between selected significantly enriched GO terms, including catalytic activity, cytoplasm, cell development, cellular localization, anatomical structure development, nervous system development, cell differentiation, and neurogenesis, and their corresponding candidate target genes. (**B**) KEGG chord diagram showing the relationships between representative enriched KEGG pathways, including protein digestion and absorption, pancreatic secretion, phagosome, bile secretion, and aldosterone synthesis and secretion, and their corresponding candidate target genes. Candidate target genes are shown on the left, with the top 20 genes displayed by default and ranked according to the number of enriched GO terms or KEGG pathways with which they are associated. Enriched GO terms or KEGG pathways that passed the significance threshold are shown on the right and ranked from the smallest to the largest *p*-value. Links indicate that a target gene is associated with the corresponding GO term or KEGG pathway.

GO enrichment analysis of these target genes covered the three major categories of Biological Process, Cellular Component, and Molecular Function. The greatest number of annotated terms was found in the Biological Process category, with major enrichment in cell development (GO:0048468), cellular localization (GO:0051641), and anatomical structure development (GO:0048856). Within the Cellular Component category, enrichment was mainly observed in cytoplasm (GO:0005737), cell projection (GO:0042995), and cell periphery (GO:0071944). The Molecular Function category contained the fewest enriched terms and was mainly represented by catalytic activity (GO:0003824), catalytic activity acting on protein (GO:0140096), and protein binding (GO:0005515) (Figure 4A and Figure 5A). KEGG enrichment analysis showed that the targets were distributed among four major categories: Cellular Processes, Environmental Information Processing, Metabolism, and Organismal Systems. Enrichment was most prominent in Organismal Systems, including pathways such as protein digestion and absorption (map04974), pancreatic secretion (map04972), and bile secretion (map04976). Within the Metabolism category, the major enriched pathway was the citrate cycle (TCA cycle) (map00020), whereas within Environmental Information Processing, the Hippo signaling pathway (map04390) was prominent. Cellular Processes contained the fewest enriched pathways, mainly lysosome (map04142) and phagosome (map04145) (Figure 4B and Figure 5B).

Figure 4C,D provide a direct comparison of enrichment patterns across different groups (L1_vs_L4, L1_vs_L7, L4_vs_L7, and All). Specifically, GO terms related to development and morphogenesis, including cell development, system development, anatomical structure development, and cell differentiation, were overall more strongly enriched in L1_vs_L4 and L4_vs_L7 but relatively weaker in L1_vs_L7, suggesting that the target genes of differentially expressed miRNAs may regulate developmental remodeling more specifically during transitions between adjacent instars. Meanwhile, terms associated with membrane structure and cellular localization, such as plasma membrane and cell periphery, were more strongly enriched in L4_vs_L7. In KEGG analysis, pathways such as lysosome, protein digestion and absorption, and pancreatic secretion were significantly enriched in multiple comparison groups, indicating that these represent common features across instar transitions, whereas bile secretion was more strongly enriched in L1_vs_L4 only.

## 3. Discussion

Limited RNAi efficiency remains a major constraint on the practical application of RNA-based technologies in the control of agricultural and forestry pests. Accordingly, identifying effective strategies to improve RNAi efficiency and clarifying the underlying mechanisms have remained central concerns in this field. In Lepidoptera, Diptera, and other insect groups, variable sensitivity to dsRNA is associated with pronounced differences in dsRNA degradation, cellular uptake, and intracellular transport, which often result in unstable or reduced RNAi efficiency [18,21]. By contrast, miRNA-mediated RNAi has shown promising application potential in some pests that respond poorly to conventional dsRNA. Previous studies have shown that transgenic tobacco expressing the endogenous *Helicoverpa armigera* miR-24 caused approximately sixfold higher larval mortality within a certain period than in control plants, while leaf damage was reduced to only one-third to one-quarter of that in the controls [37]. In *Chilo suppressalis*, transgenic rice expressing Csu-novel-miR15 significantly inhibited larval growth [38]. In addition, plin-amiR technology, which was designed on the basis of an insect pre-miRNA backbone, increased larval mortality in *H. armigera* from approximately 22% in the wild-type control to 50–60%, accompanied by evident growth retardation, pupation failure, and phenotypic defects [39]. More recent studies have further shown that insect-derived miRNAs are not limited to regulating insect growth and development. For example, the salivary miR-7-5P secreted by *Nilaparvata lugens* can enter host rice plants and target multiple plant immunity-related genes, including *OsbZIP43*, thereby suppressing plant immunity and facilitating feeding [27]. Together, these findings indicate that miRNAs have important biological regulatory functions and may also be informative for future pest-management-oriented studies. Nevertheless, the progression from candidate identification to practical RNA-based intervention requires direct validation of miRNA function, target interactions, delivery feasibility, and biosafety under species-specific contexts [11,12].

At present, however, research on miRNAs in *H. cunea* remains limited. To systematically characterize the dynamic expression patterns of miRNAs across larval instars in this species, the present study constructed miRNA libraries from 1st-, 4th-, and 7th-instar larvae for the first time and analyzed their expression characteristics in a systematic manner. After sequencing quality control (Table 1), the numbers of deduplicated unique reads ranged from 586,869 to 1,078,229, and genome-mapping rates ranged from 48.79% to 84.55%, values that are broadly comparable to those reported in miRNA studies of insects such as *Bemisia tabaci* [40] and *Hermetia illucens* [41]. Notably, both sequencing depth and mapping rates varied among libraries. Based on the per-library category summaries (Tables S2 and S3), this variation was driven primarily by differences in the fraction of unmapped reads, whereas annotated categories such as rRNA and tRNA were consistently detected across libraries and did not alone account for the full range of mapping-rate differences. We infer that these technical differences were unlikely to have substantially altered the major stage-associated expression patterns observed in this study. Further analysis showed that the miRNA sequence lengths of all three instars displayed major peaks at 18 nt and 20 nt (Figure 1A), which is generally consistent with reports that the main miRNA size distribution in insects such as *Helicoverpa armigera* [42] and *Coccinella septempunctata* [43] falls within 18–26 nt. Because Figure 1A represents the overall size distribution of post-filtering small-RNA reads rather than annotated mature miRNAs only, the prominent 18-nt peak should not be interpreted as the canonical mature miRNA size. Notably, clear peaks were also observed at 20 nt in all three instars and at 22 nt in the 1st instar [44], whereas no prominent peak appeared in the 26–31 nt range typically associated with piRNA-sized small RNAs [26]. This study substantially expands the miRNA resource of *H. cunea* and provides a data foundation for the subsequent screening of functional miRNAs and the development of miRNA-based RNAi technologies.

Through bioinformatic analysis, a total of 1080 known miRNAs and 587 bioinformatically predicted novel miRNAs were identified from the three miRNA libraries (1st-, 4th-, and 7th-instar larvae). Compared with related studies in insects such as *Bombyx mori* [45] and *Apis mellifera* [46], the number of miRNAs identified here was relatively high, which may be related to the still incomplete annotation of the *H. cunea* genome. Notably, 349 known miRNAs and 196 bioinformatically predicted novel miRNAs were continuously expressed across all three instars, which is consistent with the possibility that some of these miRNAs may be associated with basal regulatory processes across larval development. Among these shared miRNAs, animal-miR-2766 and novel-m0029-3p were the most highly expressed known and bioinformatically predicted novel miRNAs, respectively. Given that homologous miR-2766-3p has been implicated in metamorphosis-related regulation in other lepidopteran insects [47], its high expression in *H. cunea* suggests a possible developmental relevance.

Analysis of differentially expressed miRNAs provided further clues for understanding stage-associated transcriptional changes across larval instars in *H. cunea*. Because the present study is based on expression profiling and bioinformatic prediction, functional interpretations should be regarded as hypothesis-generating rather than definitive evidence of miRNA function in *H. cunea*. Different numbers of differentially expressed miRNAs were identified in pairwise comparisons among the three developmental stages, with the largest number detected in L1_vs_L7, indicating that the transition from early to late larval instars is accompanied by more pronounced shifts in miRNA expression. In all pairwise comparisons, the number of upregulated miRNAs exceeded that of downregulated miRNAs, a pattern that is broadly consistent with observations reported in other insects, such as *Sitobion miscanthi* [48], and may reflect increased divergence in post-transcriptional regulation across development. In the present study, however, our data directly support only the differential expression of these miRNAs across larval instars of *H. cunea*. Functional interpretations remain tentative and are based primarily on studies of homologous miRNAs in other insect species. In the comparison between 1st- and 4th-instar larvae, animal-miR-4448 showed the largest decrease, whereas animal-miR-9186p showed the largest increase in expression (Figure 3A). In other insects, homologous miR-4448 has been associated with pyrethroid resistance in *Culex pipiens pallens* [49] and antiviral responses in *Aedes albopictus* [50]. In the comparison between 1st- and 7th-instar larvae, animal-miR-993-3p showed the largest decrease, whereas animal-let-7-5p_7 showed the largest increase in expression (Figure 3B). In *Nilaparvata lugens*, homologous miR-993-3p has been reported to target the detoxification-related P450 gene *CYP6ER1* and thereby influence insecticide sensitivity [51,52]. In *Bombyx mori*, let-7 has been linked to the regulation of late larval development and hormone biosynthesis [32]. In the comparison between 4th- and 7th-instar larvae, animal-miR-11987 showed the largest decrease, whereas animal-miR-16b-5p_4 showed the largest increase in expression (Figure 3C). These cross-species findings provide a useful context for interpreting the expression patterns observed here, but they do not establish equivalent functions in *H. cunea*. Therefore, the stage-dependent expression of these miRNAs in our dataset should be viewed primarily as evidence for their potential relevance to larval development, whereas their precise biological roles in *H. cunea* remain to be resolved through direct functional validation.

To further clarify the potential regulatory genes and functions of the differentially expressed miRNAs, we performed target-prediction analysis. The 52 differentially expressed miRNAs were predicted to target 16,784 genes, a number far exceeding that of the miRNAs themselves. This pattern is not uncommon in insects. For example, in *Nilaparvata lugens* [44], the number of target genes has been reported to be 338 times greater than the number of miRNAs, and similar trends have been observed in other insects. These results suggest that a single miRNA may be associated with multiple target genes and thereby participate in the control of different functional modules. Conversely, the same key pathway or gene set may also be jointly regulated by multiple miRNAs. GO enrichment results further showed that the potential regulation mediated by these differentially expressed miRNAs has clear developmental relevance (Figure 4A,C). Target genes were mainly enriched in Biological Process terms such as cell development, anatomical structure development, and cellular localization, suggesting that these miRNAs may participate in tissue formation and structural remodeling across larval instars, while also influencing developmental progression through processes such as protein localization and intracellular transport. Combined with the enrichment of cytoplasm and protein binding in the Cellular Component and Molecular Function categories, these results suggest that miRNA-mediated regulation may act preferentially on cytoplasmic and membrane-associated functional nodes, thereby affecting enzymatic reactions and protein-interaction networks. KEGG enrichment analysis further linked the potential functions of miRNAs to specific metabolic pathways and signaling networks (Figure 4B,D). Enrichment of target genes in pathways such as protein digestion and absorption suggests that miRNAs may be associated with adaptation to stage-specific nutritional demands. Enrichment in the TCA cycle is consistent with the possibility that central energy metabolism is differentially regulated across larval instars. Likewise, the enrichment of development-related signaling pathways such as the Hippo signaling pathway provides a plausible framework for interpreting the developmental expression patterns observed here. Taken together, these results suggest that miRNAs are likely associated with larval developmental regulation in *H. cunea* larvae; however, the specific functional contributions of individual miRNAs and pathways remain to be established through direct experimental validation. Accordingly, the candidate miRNAs identified in the present study should not be interpreted as ready-to-use pest-control molecules, but rather as hypothesis generating candidates that warrant further functional and applied evaluation.

## 4. Materials and Methods

### 4.1. Insects and Sample Collection

*Hyphantria cunea* larvae were obtained from a laboratory colony maintained at Zhejiang A&F University (geographical coordinates: 30°15′28′′ N, 119°43′34′′ E). The colony was continuously reared for multiple generations in an artificial climate chamber under standardized conditions: 25 ± 2 °C, 70% ± 5% relative humidity, and a 14 h light/10 h dark (14L:10D) photoperiod. Larvae were fed ad libitum with fresh mulberry leaves (*Morus alba*) collected from the Zhejiang A&F University campus. The leaves were replaced daily, and old or deteriorated leaves were discarded to minimize variation in feeding quality. For miRNA analysis, whole-body samples were collected from larvae at three representative developmental stages: the 1st, 4th, and 7th instars (hereafter L1, L4, and L7). These stages specifically encompass the typical aggregation phase (L1), the behavioral transition phase (L4), and the dispersal phase (L7), thereby covering the critical developmental window of the larval aggregation–dispersion transition. Given the substantial differences in body size among larval instars, the number of individuals included in each biological replicate was adjusted to ensure sufficient sample input. Accordingly, each 1st instar biological replicate consisted of 50 larvae, and each biological replicate of the 4th and 7th instars consisted of 10 larvae. To reduce potential variation arising from recent feeding, larvae were subjected to a short period of starvation prior to collection. Samples were harvested immediately, snap-frozen in liquid nitrogen, and subsequently stored at −80 °C until RNA extraction.

### 4.2. RNA Extraction, Library Construction, and Sequencing

Total RNA extraction, miRNA library construction, and high-throughput sequencing were performed by Shanghai Personal Biotechnology Co., Ltd. (Shanghai, China). Small RNA (sRNA) libraries were prepared using the QIAseq miRNA Library Kit (QIAGEN, Hilden, Germany) according to the manufacturer’s instructions. For each library, 100 ng of total RNA was used as input for small RNA library construction. Briefly, adapter ligation, reverse transcription, and PCR amplification were performed to generate the final sRNA libraries. After adapter ligation and amplification, the resulting library fragments were approximately 147 bp, which is consistent with the expected size range of QIAseq miRNA libraries.

Library quality was assessed using an Agilent 2100 Bioanalyzer with the Agilent High Sensitivity DNA Kit (Agilent Technologies, Santa Clara, CA, USA). Library concentration was quantified using the Quant-iT PicoGreen dsDNA Assay Kit (Invitrogen, Carlsbad, CA, USA) on a QuantiFluor-ST fluorometer (Promega, Madison, WI, USA), and the effective library concentration was further validated by qPCR using a StepOnePlus Real-Time PCR System (Thermo Fisher Scientific, Waltham, MA, USA). Following normalization, libraries were pooled in equimolar amounts and sequenced on an Illumina platform using a PE150 (paired-end 150 bp) configuration. Although mature miRNAs are typically ~22 nt in length, the PE150 setting was used as part of the standardized sequencing workflow of the service provider. Importantly, only Read 1 (R1) was used for downstream miRNA identification and quantification, whereas Read 2 (R2) was excluded from the miRNA-calling workflow. Because the final library fragments were approximately 147 bp, the 150-bp R1 read length was sufficient to traverse the insert-derived small-RNA sequence together with the flanking adapter-derived sequence required for reliable adapter trimming and subsequent small-RNA recovery.

### 4.3. Data Quality Control

The image files generated by the sequencing platform were converted into raw data in FASTQ format using the platform software. Quality control and primary preprocessing of the raw reads were performed by Shanghai Personal Biotechnology Co., Ltd. using an in-house preprocessing script rather than fastp. Specifically, the specific 3′ adapter sequences in Read 1 (R1; AACTGTAGGCACCATCAAT) were removed from the raw reads, followed by quality trimming based on sequence quality. A sliding-window approach with a window size of five nucleotides was applied, and when the average Phred quality score within a window was lower than 20, the sequence was truncated from the beginning of that window and the downstream low-quality portion was discarded.

After adapter removal and quality trimming, only reads with lengths of 18–36 nt were retained as clean reads for downstream analysis. Here, the 18–36 nt size selection refers to the length of the small-RNA insert after adapter removal rather than the original raw read length. Reads shorter than 18 nt after trimming were discarded, thereby removing extremely short fragments and likely adapter–adapter products, whereas reads longer than 36 nt were excluded to reduce interference from longer non-coding RNAs or degradation-derived fragments. Identical sequences within each sample were then collapsed and counted as unique reads for subsequent analyses. Detailed preprocessing statistics are provided in Table S1.

### 4.4. Sequence Alignment and miRNA Annotation

Genome alignment was performed using miRDeep2 (v2.0), with the mapper.pl module invoking Bowtie (v2.5.1) to map clean reads to the *H. cunea* reference genome. Precursor and mature miRNA sequences from related species were retrieved from miRBase (v22) to serve as templates for annotating known miRNAs. Given that *H. cunea* is not yet represented in miRBase, a comprehensive animal miRNA database was utilized for homology-based identification to enhance annotation coverage and accuracy. For sequences that remained unassigned to any known RNA categories after genome alignment and annotation, putative novel miRNA candidates were predicted using mireap (v0.2) based on the characteristic secondary structures of miRNA precursors. In this study, miRDeep2 was used only for read mapping to the reference genome and was not used for novel miRNA prediction. Novel miRNA candidates were predicted separately using mireap. To resolve potential overlaps in small RNA (sRNA) functional categories, a hierarchical annotation strategy was implemented. Each sRNA was assigned a unique functional identity according to the following priority: known miRNA > piRNA > rRNA > tRNA > snRNA > snoRNA > repeat > exon/intron > novel miRNA. Finally, the distribution and abundance of all sRNA biotypes across different developmental stages were summarized.

### 4.5. miRNA Expression Quantification and Differential Expression Analysis

The abundance of mature and novel miRNAs was quantified using HTSeq (v0.9.1) based on the number of reads aligned to each sequence. To facilitate downstream comparisons of expression levels across miRNAs and samples, raw read counts were normalized as Transcripts Per Million (TPM). Differential expression analysis was subsequently performed using DESeq2 (v1.38.3), which accounts for differences in library size through internal normalization of count data before statistical testing. Based on the DESeq2 statistical framework, miRNAs exhibiting a |log_2_(fold change)| > 1 and a *p*-value < 0.05 were identified as significantly differentially expressed miRNAs (DEMs). Raw *p*-values were adjusted for multiple testing using the Benjamini–Hochberg (BH) method. Bidirectional hierarchical clustering of differentially expressed miRNAs and samples was performed based on expression values, using Euclidean distance and complete linkage.

### 4.6. Target Prediction and Enrichment Analysis of Differentially Expressed miRNAs

Target genes of the identified differentially expressed miRNAs were predicted using a combination of MiRanda (v3.3a), RNAhybrid (v2.1.2), and TargetScan (v8.0). To enhance the reliability of the screening process, the 3′UTR sequences of *H. cunea* mRNAs were utilized as target templates for alignment with the mature miRNA sequences. For the predicted target gene set, functional annotation and pathway enrichment were conducted using clusterProfiler (v4.6.0). Gene Ontology (GO) and Kyoto Encyclopedia of Genes and Genomes (KEGG) analyses were implemented to elucidate the biological significance of these targets. Enrichment significance was determined using the hypergeometric test, with a *p*-value < 0.05 set as the threshold for significant enrichment. Furthermore, enrichment analyses were performed independently for the overall differentially expressed miRNAs, as well as for the upregulated and downregulated subsets, to identify specific biological processes and functional pathways potentially modulated by these miRNAs during larval development.

## 5. Conclusions

In summary, this study presents the first comprehensive miRNA expression dataset spanning larval instars in *H. cunea* and identifies candidate miRNAs with stage-associated expression patterns. The predicted target genes and enrichment results suggest that miRNAs may be linked to developmental and metabolic processes during larval ontogeny. Because the present work was intended to provide a sequencing-based resource and candidate framework, independent experimental validation of the predicted novel miRNA candidates remains a priority for future investigation. These findings therefore provide a useful foundation for future functional analyses and may inform subsequent exploration of RNAi-based management strategies for *H. cunea*.

## Figures and Tables

**Table 1:** An overview of sRNA-seq datasets.

Sample	Raw Reads	Clean Reads (%)	Mapped Reads (%)
L1_1	14,927,824	7,233,279 (48.00%)	4,274,620 (59.10%)
L1_2	30,978,208	18,113,019 (58.00%)	10,022,249 (55.33%)
L1_3	15,973,602	9,341,463 (58.00%)	4,557,974 (48.79%)
L4_1	15,454,540	8,097,775 (52.00%)	6,846,924 (84.55%)
L4_2	15,475,532	7,341,611 (47.00%)	4,032,860 (54.93%)
L4_3	16,405,506	6,689,861 (40.00%)	3,700,902 (55.32%)
L7_1	16,284,405	9,106,967 (55.00%)	5,931,025 (65.13%)
L7_2	16,339,754	8,783,918 (53.00%)	6,341,978 (72.20%)
L7_3	18,623,270	8,518,726 (45.00%)	6,210,058 (72.90%)

**Table 2:** Top 10 known miRNAs and their sequences identified in three larval small RNA libraries of *Hyphantria cunea*.

Known miRNA	Sequence (5’-3’)	Expression Level (TPM)	miRNA Family	Pre-miRNA
animal-miR-2766	UCAGUCUUGUCGAAUGGUGGGU	1,236,033.7090	mir-2766	GAACCUGAAGAAGUCAAAACGCACUACUCUUCCACCGUUCGUCUCGACUGGCGCCGUCCCACGCAUCAGUCUUGUCGAAUGGUGGGUGAGUUCUGCGUA
animal-miR-276-3p_2	UAGGAACUUCAUACCGUGCUCU	1,227,090.9280	mir-276	GGUGACUGCCAUCAGCGAGGUAUAGAGUUCCUACGGUAAUCGAUUGAAACUUUGUAGGAACUUCAUACCGUGCUCUUGGAUAGCCGUUUACC
animal-miR-1	UGGAAUGUAAAGAAGUAUGGAG	779,488.1899	mir-1	CGAAAGUUCCAUGCUUCCUUGCAUUCAAUAGUGAUUCUGAAAGCAUAUGGAAUGUAAAGAAGUAUGGAGCGCUCGG
animal-miR-278-3p_2	UCGGUGGGAUCUUCGUCCGUUU	691,913.5274	mir-278	GUAUCAAGUGCUACGCCCGGACGAACUUCCCAGCUCGGCCGACAAUCAGGGGUCGGUGGGAUCUUCGUCCGUUUGUAUCGCUUAUAC
animal-miR-750_5	CCAGAUCUAUCUUUCCAGCUCA	652,211.8589	mir-750	UCCAGUAAUAUUACAGGCCUCACGUCUGAGUUGGACAGGGGAUCUUGACAGUUUAAUGUCACUUGCUGCCAGAUCUAUCUUUCCAGCUCACGCGUGAGGCCG
animal-bantam-3p_2	UGAGAUCAUUGUGAAAGCUGAUU	351,298.7062	bantam	UCGAAAACGAAACUGGUUUUCACAAUGAUUUGACAGAUAGAUUCGAUUCUGAGAUCAUUGUGAAAGCUGAUUUUGUUGAAAAGUCGA
animal-miR-2755-3p	CACCCUGUCAGACCAUACUUGUU	251,039.0446	mir-2755	UUUGGUGAAGCAAGGUGGCCUAGCAGAGUGUUUUCAACAAAGCUUGCACCCUGUCAGACCAUACUUGUUUUAACUAACC
animal-miR-8-5p	CAUCUUACCGGGCAGCAUUAGA	210,615.1555	mir-8	GUCUGUUCACAUCUUACCGGGCAGCAUUAGAUAUUUUUGAAAUACUUCUAAUACUGUCAGGUAAAGAUGUCGUCCGAAC
animal-miR-9-5p_5	UCUUUGGUUAUCUAGCUGUAUGAGU	199,663.9718	mir-9	UUGGUUGUUAUCUUUGGUUAUCUAGCUGUAUGAGUGGUGUGGAGUCUUCAUAAAGCUAGAUAACCGAAAGUAAAAAUAACCC
animal-miR-14	UCAGUCUUUUUCUCUCUCCUAU	197,633.3909	mir-14	GUGGAAUUACACCUCAACGAAUUUGAACUCUGCUCUACCCGAUAAGCCUGUGGGAGCGAGAUUAAGGCUUGCUGGUCAUUUAUUACACUCGAAGUCAGUCUUUUUCUCUCUCCUAUUGGUAUAACGGUGUUUCAACGAGUCCGAGUUCCCGCACCGUAGUCAUCCACAUCAUC

**Table 3:** Top 10 bioinformatically predicted novel miRNAs and their sequences predicted from three larval small RNA libraries of *Hyphantria cunea*.

Novel miRNA	Sequence (5’-3’)	Expression Level (TPM)	Location	Pre-miRNA	MFE (Pre-miRNA)
novel-m0029-3p	CUUUGCCCUCAGAAACUGAAAC	538,248.6600	Chr01:23542832–23542853:+	AUAUACGUUUUCGGUUUUGAGGGUGAAACUAGUUUACAAUCUCAGCUUUGCCCUCAGAAACUGAAACAACGUGUUAU	−28.50
novel-m0026-3p	CUUUGCCCUCAGAAACUGAAAC	485,665.2335	Chr01:23542068–23542089:+	AUAUACGUUUUCGGUUUUGAGGGUGAAACUAGUUUACAAUCUCAGCUUUGCCCUCAGAAACUGAAACAACGUGUUAU	−28.50
novel-m0034-3p	CUUUGCCCUCAGAAACUGAAAC	472,158.7076	Chr01:23544190–23544211:+	AUAUACGUUUUCGGUUUUGAGGGUGAAACUAGUUUACAAUCUCAGCUUUGCCCUCAGAAACUGAAACAACGUGUUUA	−28.50
novel-m0021-3p	CUUUGCCCUCAGAAACUGAAAC	469,803.3360	Chr01:23540800–23540821:+	UUAUACGUCUUCGGUUUUGAGGGUGAAACUAUUUUAUAGUCUCAGCUUUGCCCUCAGAAACUGAAACAACGUGUUAU	−26.70
novel-m0025-3p	CUUUGCCCUCAGAAACUGAAAC	453,156.1439	Chr01:23541815–23541836:+	UUAUACGUCUUCGGUUUUGAGGGUGAAACUAUUUUAUAGUCUCAGCUUUGCCCUCAGAAACUGAAACAACGUGUUAU	−26.50
novel-m0039-3p	CUUUGCCCUCAGAAACUGAAAC	446,965.2958	Chr01:23545540–23545561:+	AUAUACGUUUUCGGUUUUGAGGGUGAAACUAGUUUACAAUCUUAGCUUUGCCCUCAGAAACUGAAACAACGUGUUUA	−28.60
novel-m0024-3p	CUUUGCCCUCAGAAACUGAAAC	445,134.6892	Chr01:23541557–23541578:+	AAAUACGUUUUCGGUUUUGAGGGUGAAACUAGUUUAAAAUCUCAGCUUUGCCCUCAGAAACUGAAACAACGUGUUAU	−29.90
novel-m0023-3p	CUUUGCCCUCAGAAACUGAAAC	416,782.7378	Chr01:23541304–23541325:+	AAAUACGUUAUCGGUUUUGAGGGUGAAACUAGUUUUCAAUAUCAGCUUUGCCCUCAGAAACUGAAACAACGUGUUGU	−27.90
novel-m0032-3p	CUUUGCCCUCAGAAACUGAAAC	408,215.9975	Chr01:23543684–23543705:+	AUAUACGUUUUCGGUUUUGAGGGUGAAACUUGUUUACAAUCUCAGCUUUGCCCUCAGAAACUGAAACAACGUGUUAU	−26.70
novel-m0036-3p	CUUUGCCCUCAGAAACUGAAAC	399,279.5407	Chr01:23544695–23544716:+	AUAUACGUUUUCGGUUUUGAGGGUGAAACUAGUUUACAAUCUCAGCUUUGCCCUCAGAAACUGAAACAACGUGUUUA	−28.50

## Data Availability

Small RNA-seq data generated from the 1st-, 4th-, and 7th-instar larvae of the fall webworm (*Hyphantria cunea*) have been deposited at NGDC (National Genomics Data Center) at GSA under accession number PRJCA062756 and are publicly available as of the date of publication. The datasets generated and analyzed during the current study are available from the corresponding authors on reasonable request.

## Supplementary Materials

The following supporting information can be downloaded at: https://www.mdpi.com/article/10.3390/ijms27093884/s1.

## Abbreviations

The following abbreviations are used in this manuscript:

miRNA	microRNA
siRNA	small interfering RNA
piRNA	Piwi-interacting RNA
dsRNA	double-stranded RNA
RNAi	RNA interference
UTR	Untranslated region
TPM	transcripts per million
